# Hepatitis C Virus Cascade of Care After the Introduction of Direct-Acting Antiviral Medications in Suriname

**DOI:** 10.7759/cureus.86873

**Published:** 2025-06-27

**Authors:** Anfernee K Neus, Meerte S Macdonald, Soeradj Harkisoen, Lycke Woittiez, Cheryll B Monsanto, S V Jarbandhan, Karin Waldring, Jimmy Roosblad, Stephen G Vreden

**Affiliations:** 1 Medicine, Anton de Kom University of Suriname, Paramaribo, SUR; 2 Medical Microbiology, Amsterdam University Medical Center, Amsterdam, NLD; 3 Microbiology, University Medical Center Utrecht, Utrecht, NLD; 4 Infectious Diseases, Academic Hospital Paramaribo, Paramaribo, SUR; 5 Pharmacy, Academic Hospital Paramaribo, Paramaribo, SUR; 6 Gastroenterology, Academic Hospital Paramaribo, Paramaribo, SUR; 7 Infectious Diseases, St. Vincentius Hospital, Paramaribo, SUR; 8 Clinical Chemistry, Academic Hospital Paramaribo, Paramaribo, SUR; 9 Internal Medicine, Foundation for the Advancement of Scientific Research in Suriname, Paramaribo, Suriname

**Keywords:** aspartate aminotransferase-to-platelet ratio index (apri score), direct-acting antiviral (daa), hepatitis c virus (hcv) infection, sofosbuvir (sof), suriname, velpatasvir

## Abstract

Introduction: Suriname has a hepatitis C virus (HCV) prevalence of 1%. In 2019, direct-acting antivirals (DAAs) were introduced. Treatment was started in HCV patients who could afford the procurement of DAAs, since no insurance policy covered treatment at that time. This study aims to identify barriers in our HCV treatment cascade and evaluate the aspartate aminotransferase-to-platelet ratio index (APRI) as a non-invasive test for detecting fibrosis and cirrhosis.

Materials and methods: We developed an HCV cascade of care for patients in the four general hospitals in the capital, spanning from linkage to care through to resolution of the infection. Furthermore, liver elastography is a crucial diagnostic tool for staging liver disease. Unfortunately, this is not widely available and can delay treatment initiation. Therefore, we evaluated the APRI as an alternative tool for staging liver disease.

Results: Of the 274 HCV patients, 216 (78.8%) could be linked to care, 174 (63.9%) were eligible for treatment, 156 (57.3%) started treatment, of which 149 (54.7%) completed it. Ultimately, 123 (45.3%) individuals attended their outcome evaluation, all of whom had achieved a sustained virological response 12 weeks after treatment completion. For detecting cirrhosis, an APRI value > 1.0 had a sensitivity and specificity of 75% and 85.7%, respectively. For detecting fibrosis or cirrhosis, an APRI value > 0.7 had a sensitivity and specificity of 69.2% and 61.4%, respectively.

Conclusions: Although treatment with DAAs in Suriname is highly efficacious, several hurdles, including improving linkage and access to treatment, must be addressed to reduce the disease burden of HCV in Suriname successfully.

## Introduction

Viral hepatitis is a significant cause of morbidity and mortality worldwide. Hepatitis C virus (HCV) frequently causes chronic infections, which can ultimately lead to cirrhosis and hepatocellular carcinoma (HCC) [1]. In 2015, the global HCV prevalence was estimated at 71 million people, with approximately 1.19 million deaths due to cirrhosis and HCV [2]. However, after the introduction of direct-acting antivirals (DAAs) in 2011, the global elimination of HCV seems feasible [3]. The treatment success rate, measured by the sustained virological response 12 weeks after treatment completion (SVR12), has increased significantly compared to previous interferon-based therapies. Treatment with DAAs results in SVR12 of over 90% and is associated with a reduced risk of liver-related and overall mortality [4,5]. Although DAAs are highly effective, access to treatment remains a challenge due to their high cost, particularly in low- and middle-income countries (LMICs) and upper-middle-income countries (UMICs) [6].

In Suriname, a UMIC in South America, the overall HCV prevalence is estimated at 1.0% and mainly affects males, older generations, and individuals with Javanese heritage. HCV genotype 2 is the predominant circulating genotype [7,8]. Although Suriname is classified as a UMIC, primarily due to its abundance of natural resources [9], approximately one in five people live below the poverty line [10]. However, Suriname has implemented policies to achieve universal healthcare [11]. Healthcare is generally covered by public healthcare insurance for low-income individuals and private healthcare insurance, which ensures access to private hospitals and more comprehensive coverage.

In 2016, Suriname committed to the World Health Organization’s targets to eliminate HCV, specifically to diagnose 90% of people living with HCV (PLHCV) and treat 80% of PLHCV by 2030 [2]. Therefore, in 2019, generic DAAs, sofosbuvir/velpatasvir, were introduced in Suriname. At the time, there were no guidelines for viral hepatitis, and no healthcare insurance completely covered DAA treatment.

Chronic viral hepatitis leads to liver cirrhosis, which has an increased risk of developing HCC and other liver-related complications [12]. Depending on the type of DAAs and the presence of liver cirrhosis, the duration of treatment is adjusted, and post-treatment HCC screening needs to be continued [13]. Therefore, the timely identification of cirrhosis is crucial. Liver biopsy is the gold standard for staging the severity of liver disease, but it is not commonly used due to its invasiveness [14]. In Suriname, the FibroScan is the most used non-invasive test to assess cirrhosis. However, the FibroScan is only available in one radiology clinic, which leads to treatment delay. The aspartate aminotransferase-to-platelet ratio index (APRI) is an alternative non-invasive tool for cirrhosis, which is measured using blood parameters [15,16]. The APRI is moderately accurate in detecting cirrhosis when using a cut-off value of greater than 1.0 [15,17]. For fibrosis detection, an APRI value with a cut-off value of 0.7 had a sensitivity and specificity of 77% and 72%, respectively [15].

Despite the challenges above, this study aims to identify barriers in our HCV treatment cascade and evaluate the APRI as a non-invasive test for detecting fibrosis and cirrhosis.

## Materials and methods

Study design and study population

This prospective cohort study recruited previously and newly identified anti-HCV-positive patients from the medical files of all infectious disease specialists and gastroenterologists working in the four hospitals in Paramaribo, the capital of Suriname. These four hospitals house the only HCV treatment clinics in the country. Each patient was approached personally by their specialist with a brief explanation that curative medication was available. If a patient could not be reached after three attempts or refused treatment during the consultation, no additional steps were taken, and they were excluded from this study.

Eligibility for study enrollment was based on the European Association for the Study of the Liver 2018 HCV Treatment Guidelines [13]. Treatment was recommended for eligible patients with a positive HCV-PCR who were HCV-treatment naïve or experienced, irrespective of co-infections (HBV/HIV), extrahepatic HCV manifestations, and the stage of liver disease.

Patients were not eligible for treatment if they had a negative HCV-PCR, a limited life expectancy, or were pregnant. For pregnant individuals, treatment was postponed until after delivery. Participants were recruited from April 2019 through December 2021. Sociodemographic data, including age, sex, and ethnic background, were collected, along with HCV lifetime risk factors and laboratory testing results.

Study procedure

HCV Assessment and Liver Function

All patients with a positive HCV antibody test underwent molecular diagnostic testing for HCV RNA detection. After assessment of eligibility, treatment was proposed. Patient assessment included laboratory tests for CBC, a hepatic function panel (serum albumin, total and direct bilirubin, alanine aminotransferase, aspartate aminotransferase, and alkaline phosphatase levels), and estimated glomerular filtration rate. Liver elastography was only performed to assess cirrhosis or fibrosis when available. Laboratory tests were repeated during and after treatment when possible. In addition, we calculated the APRI to determine the presence and extent of liver damage in patients from whom liver elastography results were available [16-17].

Serological Testing

Patients were screened for HIV and HBV using the Abbott Architect HIV Ag/Ab Combo assay and the Abbott Architect HBsAg Qualitative II assay, respectively (Abbott Laboratories, Chicago, Illinois, USA). The outcome of these tests did not lead to the exclusion of HCV treatment but was meant to adjust HIV treatment, where necessary, and to allow close monitoring of the HBV infection during treatment.

Molecular Diagnostic Assessment of HCV RNA, HBV DNA, and HIV RNA

Plasma HCV RNA levels were measured using the X-tail real-time HCV assay, which has a lower detection limit of 18.4 IU/ml (95% CI 15.3-24.1 IU/ml). As previously described, this X-tail RT-PCR was highly sensitive (18.4 IU/mL, 95% probit probability) and robust against genotype variation [18]. Briefly, reactions of 20 μl contained 10 μl of RNA extract, 21-step qPCRSYBR mix (Thermo Scientific Verso SYBRGreen 1-step qRT-PCR Kit), 300 nM of each primer, and 1 μl enzyme mix. Thermal cycling was performed under the following conditions: 50°C for 15 minutes, 95°C for 15 minutes, 45 cycles at 95°C for 15 seconds, 56°C for 30 seconds, and 72°C for 30 seconds, followed by a melt curve analysis. Plasma HBV DNA levels were measured in HBsAg seropositive individuals using the X-tail real-time HBV assay. Plasma HIV RNA was calculated using the Generic Biocentric assay (Biocentric, Bandol, France) [18].

APRI

The formula for calculating the APRI is:

\[ \text{APRI} = \left( \frac{\frac{\text{AST (U/L)}}{\text{Upper Limit of Normal (ULN)}}}{\text{Platelet Count } (10^9/\text{L})} \right) \times 100 \]

In a comparative study, Lin et al. found a sensitivity of 76% and a specificity of 72% for predicting cirrhosis when using a cut-off value of > 1.0 [15]. In the same study, for fibrosis detection, an APRI cut-off value of > 0.7 showed a sensitivity and specificity of 77.0% and 72%, respectively [15].

Treatment

Treatment was initiated in all eligible patients with a fixed-dose combination of sofosbuvir 400 mg and velpatasvir 100 mg, which was self-administered once daily for 12 weeks. Procurement of medication was out of pocket, and depending on the health insurance provider, it was partially or not reimbursed.

Outcome measures

The primary outcome measure was the proportion of HCV-infected patients eligible for treatment who achieved SVR12. Secondary outcomes were the proportion of patients who had not received therapy despite being eligible. Additionally, we calculated the sensitivity and specificity of the APRI for detecting cirrhosis or fibrosis, using liver elastography as a reference test.

HCV cascade of care

The treatment cascade framework encompasses individuals identified as HCV-positive, those with active HCV infections, and those with SVR12 [19-20]. The seven stages of our HCV cascade of care were (1) tested anti-HCV seropositive, (2) linked to care, (3) eligible for treatment (i.e., viral confirmation and no contraindications for treatment), (4) started with DAAs, (5) completed DAA treatment, (6) underwent posttreatment evaluation, and (7) confirmed virological response after 12 weeks.

Data collection and statistical analysis

Sociodemographic data and laboratory test results were extracted from the medical files, anonymized, and entered into a secure Excel file (Microsoft Corp., Redmond, WA, USA). For statistical analysis, RStudio version 2024.09.1+394 (Posit, PBC, Boston, MA, USA) was used.

Ethical considerations

Ethical clearance and obtaining informed consent were deemed unnecessary, as this study aimed to evaluate the current standard of HCV care in Suriname, and participants were not subjected to procedures other than those necessary to provide care for their ailment. However, the Institutional Review Board of the Academic Hospital Paramaribo approved our analysis of the data and the publication of the results (approval number: CR/LLKF/ak/no.273, approval date: May 30, 2025).

## Results

Participant characteristics

**Figure 1:**
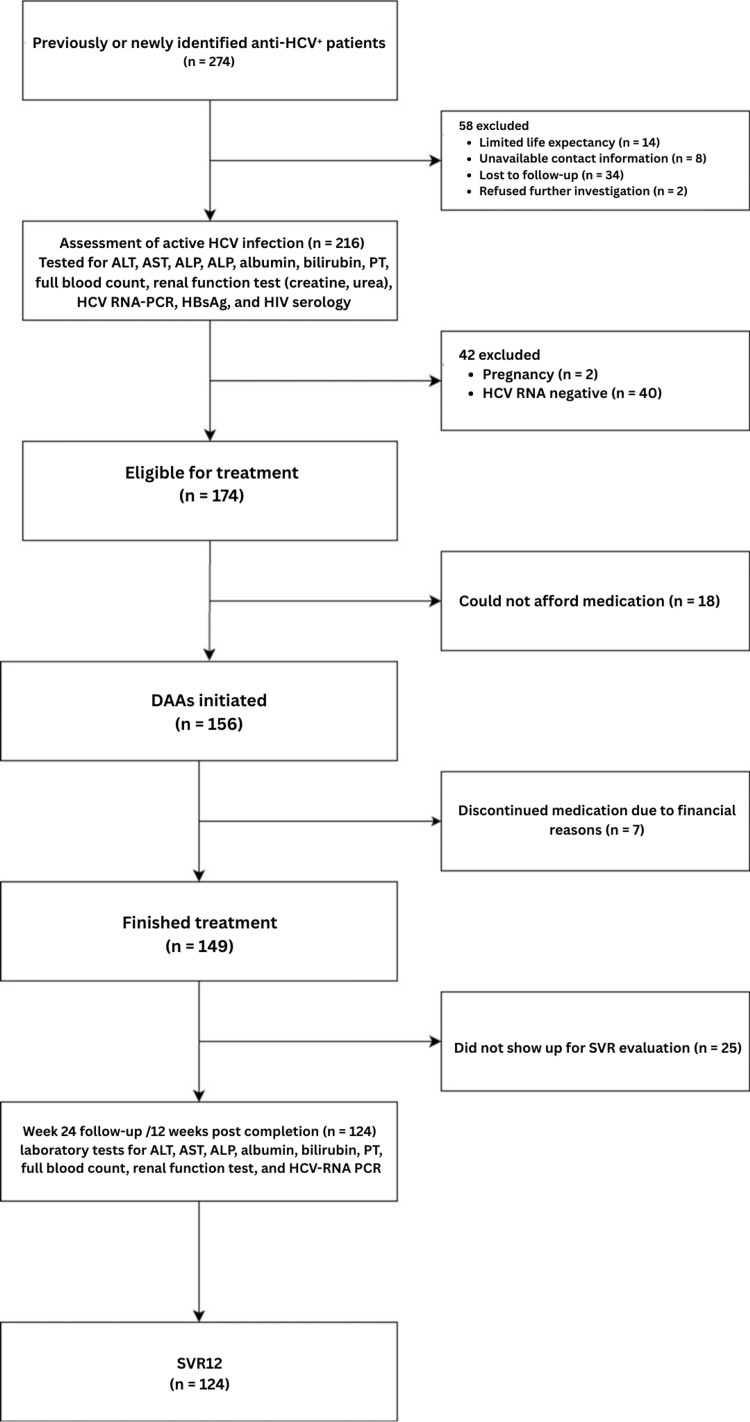
Flow diagram of the recruitment and management of a cohort of 274 patients with a known positive anti-HCV serology ALT: alanine aminotransferase, AST: aspartate aminotransferase, ALP: alkaline phosphatase, PT: prothrombin time, RNA: ribonucleic acid, PCR: polymerase chain reaction, HBsAg: hepatitis B surface antigen, HIV: human immunodeficiency virus, HCV: hepatitis C virus, SVR12: sustained virological response 12 weeks after treatment completion

Between March 2019 and December 2021, we identified 274 anti-HCV-positive patients using the flowchart described in Figure 1. Of these, eight (2.9%) could not be reached, and two refused further investigation. Thirty-four subjects (12.4%) did not attend their follow-up appointment, and 14 (5.1%) had a limited life expectancy, resulting in a total of 58 exclusions. This left 216 anti-HCV seropositive participants for inclusion in the study. Of this group, 102 (47.2%) were male, 114 (52.8%) were female, and the median age was 58 years (range: 23-85 years). Six (2.8%) had an HBV coinfection, and six (2.8%) had an HIV coinfection (Table 1).

**Table 1:** Demographic and clinical characteristics of participants eligible for treatment HIV: human immunodeficiency virus, RNA: ribonucleic acid, PCR: polymerase chain reaction, HBV: hepatitis B virus

		All patients (n=216)
Age	Years, median (IQR)	58 (50-67)
Gender	Male, n (%)	102 (47.2%)
Laboratory results		
HIV status	Positive, n (%)	6 (2.8%)
HIV-RNA PCR < 100 copies/ml, n (%)		6 (100%)
HBV status	Positive, n (%)	6 (2.8%)
Liver-elastography result		
No pathology, n (%)		123 (56.9%)
Fibrosis, n (%)		34 (15.7%)
Cirrhosis, n (%)		37 (17.1%)
Unknown, n (%)		22 (10.2%)

HCV cascade of care

**Figure 2:**
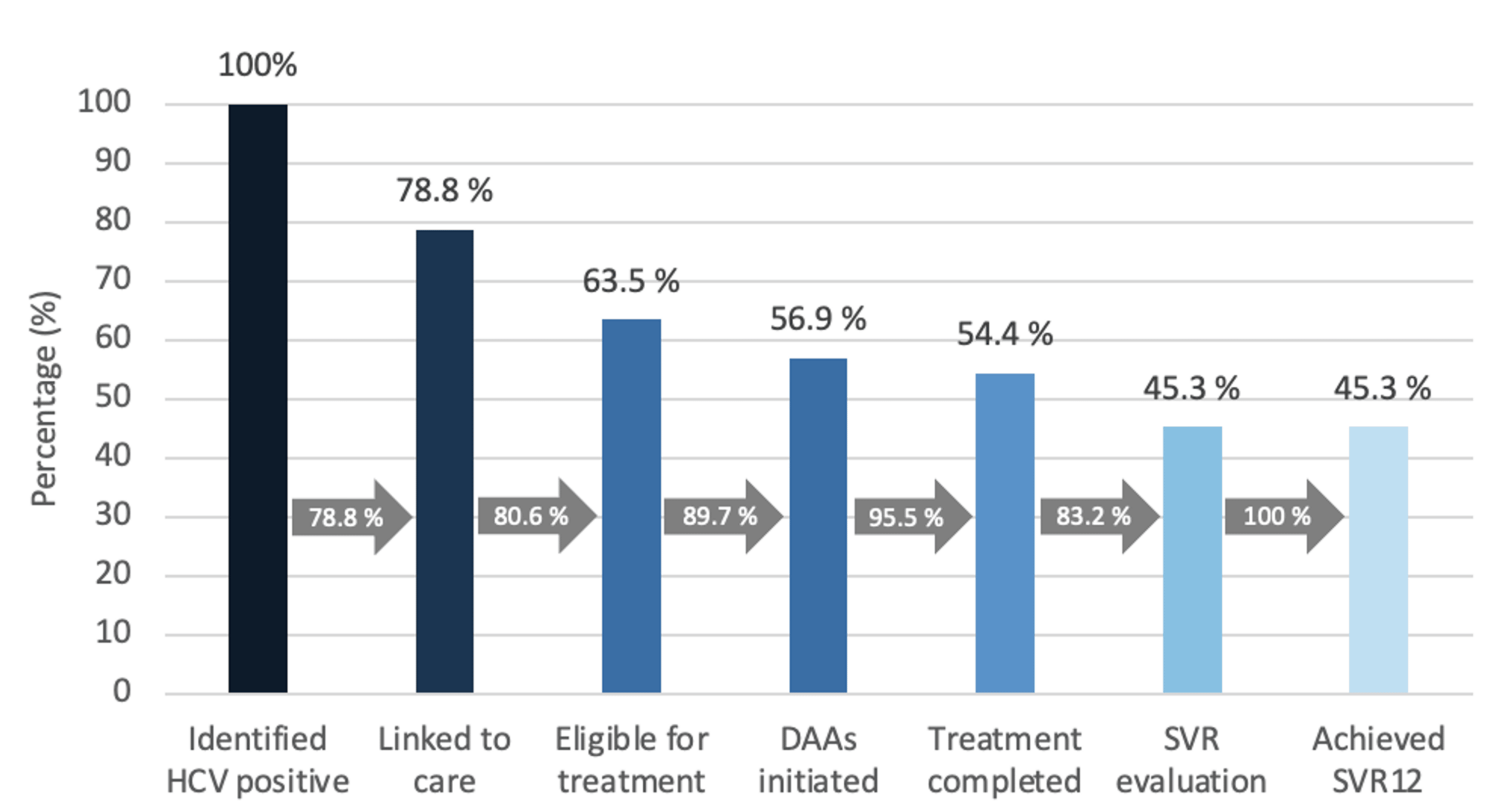
Treatment cascade of 274 anti-HCV positive patients who were recruited for (eligibility for) treatment with DAAs HCV: hepatitis C virus, DAAs: direct-acting antivirals, SVR12: sustained virological response 12 weeks after treatment completion

Of the 216 anti-HCV seropositive patients, two (0.9%) were pregnant and excluded from the study; treatment was provided after delivery. Forty (18.5%) had a negative HCV-RNA PCR and were therefore not eligible for further treatment. Overall, 156 of 174 patients (89.7%) with a treatment indication started treatment with DAAs. Eighteen (10.3%) of those eligible for treatment were unable to afford DAAs. A total of 150 (95.5%) patients who initiated DAAs completed the 12-week course. Seven patients (4.5%) were unable to complete their therapy due to financial constraints. Ultimately, 124 of 142 patients who completed the 12 weeks were tested for HCV RNA 12 weeks after DAA completion, and all achieved an SVR (Figure 2).

Liver elastography and APRI

Of the 194 patients who underwent liver elastography, 179 patients had the relevant lab values to calculate the APRI. Liver elastography detected cirrhosis in 32 (17.9%) and at least fibrosis in 65 (36.3%). Using the APRI score, 24 (13.4%) were classified as having cirrhosis, and 13 (7.3%) were classified as having fibrosis. Using the APRI for cirrhosis, the sensitivity and specificity were 75.0% and 85.7%, respectively (Table 2).

**Table 2:** Sensitivity and specificity of APRI for detecting cirrhosis in 179 patients who underwent liver elastography APRI: aspartate aminotransferase-to-platelet ratio index, CI: confidence interval

APRI	Number	Elastography no cirrhosis	Elastography cirrhosis	Sensitivity% (95% CI)	Specificity% (95% CI)
< 1.0	107	99	8	-	-
> 1.0	72	48	24	75.0% (56.6-88.5)	85.7% (73.8-93.6)
Total	179	147	32	-	-

For fibrosis detection with an APRI value > 0.7, we found a sensitivity of 69.2% and a specificity of 61.4%. The results are reported in Table 3.

**Table 3:** Sensitivity and specificity of APRI for detecting fibrosis in 179 patients who underwent liver elastography APRI: aspartate aminotransferase-to-platelet ratio index, CI: confidence interval

APRI	Number	Elastography no fibrosis/cirrhosis	Elastography fibrosis	Sensitivity% (95% CI)	Specificity% (95% CI)
< 0.7	90	70	20	-	-
> 0.7	89	44	45	69.2% (56.6-80.9)	61.4% (51.8-70.4)
Total	179	114	65	-	-

## Discussion

This study presents the outcome of our efforts to introduce DAA treatment for HCV-infected patients in Suriname. The results are reported in a cascade of care that included treatment eligibility. A significant number of anti-HCV seropositive patients (41, 15.7%) could not be reached for further evaluation or did not wish to undergo any additional testing or treatment (n = 2). A factor that may have contributed to this high dropout rate is the overlapping timeframe with the COVID-19 pandemic, which imposed movement restrictions that made access to hospital outpatient clinics challenging [21]. Furthermore, the relatively long asymptomatic course of chronic HCV may not be motivating for people to seek care. It could explain why several anti-HCV-positive patients declined further evaluation. Forty of the 216 evaluated patients (18.5%) with a positive anti-HCV test had a negative HCV-RNA result, without prior HCV treatment, and thus had spontaneously cleared the virus; this clearance rate corroborates numbers reported from Brazil (20.2%) and Canada (22.6%) [22-23].

The rate of not initiating or being unable to continue treatment due to financial constraints in our population was 14.0%, comparable to rates found elsewhere [23]. The 4.0% rate of treatment discontinuation was comparable to rates reported in the literature [23]. At the time of the study, no health insurance provider in Suriname covered the costs of DAA treatment. However, the State Health Insurance Fund had implemented a partial reimbursement policy for its clients. Fortunately, since January 2024, DAA treatment has been fully covered by all insurance companies in the country. In 78.8% of eligible patients, effective treatment could be confirmed. This result is due to the 26 patients who were not evaluated at 12 weeks post-completion, despite efforts to reach them. The figure in the last leg of the treatment cascade is lower than the 90.0% reported in a treatment effectiveness study among a socioeconomically disadvantaged cohort in the United States of America [24]. However, all patients eligible for treatment in Suriname who completed the entire HCV cascade of care achieved SVR12, a very promising result for implementing DAAs on a national scale, now that treatment is accessible to all.

The HIV positivity rate among the 216 evaluated participants, at 1.4%, is comparable to the national rate of 1.6% [25] and in line with what has been reported in Latin America and the Caribbean [26]. The rate of HBV coinfection, at 1.3%, is lower than our national infection rate, estimated to be 3.2%, and significantly lower than the range of 2-10% reported elsewhere [27,28].

​​We demonstrated SVR12 in 78.8% of patients who were eligible for treatment, which is quite similar to the 79.9% reported in a study of indigenous populations in Canada [23]. The actual rate of SVR in our population may, however, be substantially higher if we consider that 17.5% of patients who completed treatment didn’t show up for evaluation. This cause is possibly also linked to the COVID-19 pandemic [21].

The sensitivity of APRI for detecting cirrhosis is 75.0%, comparable to what has been reported elsewhere [15,29]. The specificity, however, is 85.7%, which is higher than reported by Lin [15].

For fibrosis detection, APRI had a sensitivity and specificity of 69.2% and 61.4%, respectively, which were comparable to those found by Lin [15]. This easy and inexpensive approximation method is primarily useful for identifying individuals with advanced HCV liver disease, who, even after effective treatment, should then be referred for further evaluation [30]. For that purpose, a high sensitivity is important. Despite the limited performance, we consider the APRI score to be a useful tool in guiding clinical decision-making for patients with chronic liver disease in low-resource settings.

Study limitations

This study has several limitations. First, the sample size was relatively small, which may affect the generalizability of the findings. Second, selection bias may have been introduced because previously identified HCV patients could only be recruited if they had a contact number. Third, only 82.9% of included patients had complete data for APRI calculation, which could influence the accuracy and interpretation of liver fibrosis staging results.

## Conclusions

Treatment with DAAs is, as elsewhere, highly efficacious in patients with chronic HCV infection. Overall, this study indicates the feasibility of HCV treatment with DAAs in Suriname. One of the main obstacles we observed, financial access to effective medication, has fortunately now been addressed by the availability of DAAs to all who need them. Additionally, treatment adherence proves to be quite adequate, which, together with the limited expertise required for treatment, would allow the delegation of HCV treatment to family practitioners. Such an approach may further enhance linkage to care and expand treatment access across the population.
